# Flaxseed Mucilage as a Functional Egg Replacer in the Preparation of Mayonnaise: Stability, Physicochemical, and Sensory Properties

**DOI:** 10.1111/1750-3841.71140

**Published:** 2026-05-14

**Authors:** Elif Yaver, Eda Güneş, Nursena Adıyaman

**Affiliations:** ^1^ Department of Food Processing, Vocational School of Technical Sciences Konya Technical University Selçuklu Konya Türkiye; ^2^ Department of Gastronomy and Culinary Arts, Faculty of Tourism Necmettin Erbakan University Meram Konya Türkiye

**Keywords:** egg replacer, flaxseed mucilage, mayonnaise, sustainable food, vegan diet

## Abstract

Mayonnaise is a widely consumed food dressing worldwide. Traditionally, it is prepared by emulsification of oil with egg yolk. However, the use of eggs is limited due to their cholesterol‐rich content, allergenicity, and high price, as well as the vegan and healthy dietary preferences of consumers. Flaxseed mucilage, as a natural hydrocolloid gum, exhibits comparable functional properties, especially emulsifying activity, to commercial gums. In this study, egg yolk in mayonnaise was substituted by flaxseed mucilage solution at 25%, 50%, 75%, and 100% levels to develop functional formulations. Stability, physicochemical, microbiological, textural, and sensory properties of mucilage‐added mayonnaise samples were compared with the control (100% egg yolk mayonnaise) and three different negative controls (mayonnaises made with 25%, 50%, and 75% egg yolk). Findings demonstrated that the color values of mayonnaises were considerably changed by the replacement of egg yolk with mucilage. Mayonnaise made with 100% flaxseed mucilage had a lower pH and higher moisture content than the control and negative controls. Mucilage‐added samples showed similar emulsion stability to the control. Moreover, free fatty acid and peroxide values of samples containing flaxseed mucilage were lower than those of the control and negative controls. The substitution of egg yolk with mucilage at high levels (≥ 50%) decreased hardness, adhesiveness, and gumminess values. There were no considerable alterations in microbial counts between the control and mucilage‐added samples. Samples made with 25% mucilage revealed similar acceptability scores to the control. In conclusion, flaxseed mucilage could be considered a potential egg replacer for preparing functional mayonnaise with good stability and acceptability.

**Practical Applications:**

Despite their high nutritional value, cholesterol content, allergenicity, risk of *Salmonella*, and high price are disadvantages of eggs. Flaxseed mucilage is a sustainable and natural hydrocolloid gum. It contains high levels of soluble fibers, which may exhibit positive influences on obesity, diabetes, heart diseases, and some cancer types. The development of functional mayonnaise formulations in this study by replacing egg yolk with flaxseed mucilage could represent a novel healthy alternative in the food industry.

## 1 Introduction

One of the most used sauces in the world is mayonnaise, which contains vegetable oil, egg yolk as an emulsifier, acidifying agent, sugar, salt, and spices. The estimated value of the global mayonnaise market, which was $10.8 billion in 2019, is expected to reach $13.27 billion by 2026 (Saget et al. 2021). Egg yolk, which has unique functional features, is an essential component in the preparation of mayonnaise. However, egg yolk is rich in cholesterol and a common allergen. Furthermore, the use of raw eggs could increase *Salmonella* contamination risk (Gomez et al. 2025). On the other hand, the trend of seeking out healthy food options and adopting vegan lifestyles is accelerating among consumers. The limitations of egg yolk have driven improvements in egg replacements in the food industry.

It is stated that the value of the vegan egg market reached $1.5 billion in 2021, and the increase will continue with an annual growth of 8.3% in market value until 2031 (Boukid and Gagaoua 2022). Hence, many researchers have investigated the potential of plant‐based proteins (Li et al. 2022), starches (Lu et al. 2023), and gums (Ali and el Said 2020) as egg replacers.

Nowadays, there is a growing trend of utilizing seed mucilages as natural and sustainable gums in innovative and functional food formulations. Seed mucilages like flaxseed mucilage, which are rich sources of soluble fibers, have good functional properties such as high water absorption capacity, oil absorption capacity, swelling power, solubility, and emulsifying capacity (Y. Liu et al. 2021). They have great potential in the food industry as thickeners, stabilizers, emulsifiers, suspending, film‐forming, and gelling agent because of their edible, inexpensive, sustainable, and safe features, as well as functional characteristics (Haseeb et al. 2024). Moreover, the use of mucilages in foodstuffs could reduce the risks of obesity, diabetes, cardiovascular diseases, ulcers, and cancer (Y. Liu et al. 2021).

Flaxseed mucilage can be easily obtained from whole flaxseed or seed cake by the hot‐water extraction method (Haseeb et al. 2024). The application of flaxseed mucilage to prepare functional food products was stated in some studies. In these studies, it is reported that the use of flaxseed mucilage improved textural features, technological quality, and sensory acceptance of foods (Kucka et al. 2024; Yaver 2026).

In the literature, there is limited data on the use of seed mucilages as egg replacers in mayonnaise formulations. Odep et al. (2024) showed that the replacement of egg yolk with chia seed mucilage in mayonnaise was accepted by consumers. Fernandes and Mellado (2018) found that the substitution of egg yolk with chia mucilage revealed similar stability and textural characteristics to the control. To the best of our knowledge, this is the first study on the replacement of egg yolk in mayonnaise formulation with flaxseed mucilage. Therefore, the study aimed to develop functional mayonnaise formulations by replacing egg yolk with flaxseed mucilage at 0%, 25%, 50%, 75%, and 100% levels and to investigate the influence of the substitution of egg yolk with flaxseed mucilage on the stability, physicochemical, microbiological, textural, and sensory features of mayonnaise samples. The investigated parameters were also compared with three negative controls (mayonnaises with 25%, 50%, and 75% egg yolk).

## 2 Materials and Methods

### 2.1 Materials

Cold‐pressed flaxseed cake (36.39% protein, 4.61% ash, and 28.34% total carbohydrate) was obtained from Soivera (Tokat, Türkiye). Sunflower seed oil (Çotanak, Ordu, Türkiye), chicken eggs (Bilibili, Ankara, Türkiye), red grape vinegar (Torku, Konya, Türkiye), salt, and sugar were procured from a local supermarket in Konya (Türkiye). All standards and reagents (Sigma‐Aldrich, St Louis, MO, USA) used were of analytical grade.

### 2.2 Extraction of Flaxseed Mucilage

Flaxseed mucilage (9.80% protein, 3.75% ash, 5.07% fat, and 72.31% total carbohydrate) used in this study was obtained using a conventional extraction method described in the previous studies (Yaver 2026; Yaver et al. 2026). Briefly, a mixture of flaxseed cake and distilled water (1:20, w/v) was stirred at 50°C for 3 h, followed by the filtration of the suspension. After centrifugation (Nüve NF800, Ankara, Türkiye) of the filtrate at 4300 rpm for 15 min, the supernatant was precipitated with ethanol (1:1, v/v) at 4°C for 60 min. The blend was subjected to centrifugation at 4300 rpm for 10 min. The residue was dried (Nüve KD200) at 45°C and ground in a laboratory mill (Fakir Aromatic, Vaihingen, Germany) (Yu et al. 2022).

### 2.3 Preparation of Mayonnaise Samples

Mayonnaise samples were prepared according to the method described by Ozcan et al. (2023). To prepare the control sample (containing 100% egg yolk), first, salt (3 g), vinegar (26 g), and sugar (3.5 g) were mixed with a homogenizer (ISOLAB, Wertheim, Germany) at 8000 rpm for 1 min. Following this, egg yolk (30 g) was added and homogenized at 8000 rpm for 2 min. After that, the oil (187.5 g) was added to the dispersion drop by drop during homogenization at 12,000 rpm for 15 min. The end product was stored at 4°C until further use.

To prepare negative control groups, the amount of egg yolk in the mayonnaise was reduced to 75%, 50%, and 25% individually. The procedure for making the control mayonnaise was replicated for these samples.

To prepare mayonnaise samples containing mucilage, egg yolk was replaced with flaxseed mucilage suspension (8 g mucilage/100 g distilled water) at 25%, 50%, 75%, and 100% levels. The same mayonnaise production process as in the control sample was applied to these samples.

### 2.4 Colorimetric Analysis

Chromatic coordinates on an axis for brightness (L*), green–red axis (a*), and blue–yellow axis (b*) were determined by Konica Minolta CR‐400 (Osaka, Japan). Saturation index (*SI*) [(a*^2^+b*^2^)^1/2^] and *hue* angle [arctan(b*/a*)] values were calculated with a* and b* values. Total color difference (Δ*E*) between the control and mucilage‐containing samples was determined by Equation (1).

(1) $$\begin{array}{ccc} & & \Delta E=\\ & & \;\;\;\;\sqrt{{\left(L_{\mathrm{Sample}}^{*}-L_{\mathrm{Control}}^{*}\right)}^{2}+{\left(a_{\mathrm{Sample}}^{*}-a_{\mathrm{Control}}^{*}\right)}^{2}+{\left(b_{\mathrm{Sample}}^{*}-b_{\mathrm{Control}}^{*}\right)}^{2}} \end{array}$$

### 2.5 pH and Moisture

pH measurements were recorded via a pH meter (WTW 330, Weilheim, Germany) at room temperature (25 ± 2°C) (Ünver and Çelik 2023).

Moisture content of samples was determined by the drying method in a hot air oven (Nüve KD200) at 105°C for 6 h (AOAC 2008).

### 2.6 Emulsion Stability

Five grams of mayonnaise were weighed in a Falcon tube and kept at 50°C for 24 h. At the end of the incubation, tubes were subjected to centrifugation (Nüve NF800) at 4000 rpm for 20 min. The weight of the separated phase was noted, and emulsion stability was calculated using Equation (2) (Ünver and Çelik 2023):

(2) $$\begin{array}{ccc} & & \mathrm{Emulsion}\;\mathrm{stability}\;\left(\%\right)=\left(\mathrm{Precipitated}\;\mathrm{phase}\;\mathrm{weight}/\right.\\ & & \left.\mathrm{Initial}\;\mathrm{emulsion}\;\mathrm{weight}\right)\;\times \;100 \end{array}$$

### 2.7 Oxidative Stability

Before assessing the oxidative stability of mayonnaise samples, each sample was subjected to freezing at (−24)°C for 24 h. After that, frozen samples were thawed at 25°C for 2 h to break the emulsion and centrifuged (Nüve NF800) at 10,000 rpm for 10 min (Ünver and Çelik 2021). The separated oil phase was utilized for the determination of free fatty acid (FFA) content and peroxide value of samples.

FFA content was analyzed using the AOCS method Ca5a‐40 (AOCS 2017). Briefly, 2 g of oil phase was dissolved in 25 mL of ethanol and 50 mL of diethyl ether. Approximately 0.1 N KOH solution was used for titration. FFA content of samples was measured as %oleic acid.

Peroxide value was measured according to the AOCS method Cd8b‐90 (AOCS 2017). Around 4 g of oil phase was dissolved in 10 mL of chloroform and 15 mL of glacial acetic acid. Then, 1 mL of saturated potassium iodide solution was added to the mixture. After 5 min, the mixture was titrated with 0.002 N sodium thiosulfate solution, and the results were expressed as milliequivalent oxygen per kilogram oil (meqO_2_/kg).

### 2.8 Microbiological Quality

Total aerobic mesophilic bacteria (TAMB), yeast, and molds, and total coliform bacteria counts were determined according to the methods described by Halkman (2005) and Erginkaya et al. (2016). Briefly, samples (10 g) were combined with 90 mL of 0.1% peptone water (Merck, Darmstadt, Germany) and then thoroughly mixed. Subsequent decimal dilutions were prepared from the 10^−1^ dilution. Nutrient agar (Merck), potato dextrose agar (Merck), and lauryl sulfate tryptose broth (Merck) were used for TAMB count (30°C for 72 h), total yeast and mold (25°C for 120 h), and coliform bacteria (37°C for 24 h) counts, respectively.

### 2.9 Texture

Textural evaluation of mayonnaise samples was performed via a texture analyzer (Stable Microsystems TA‐XT.plus, Surrey, UK) equipped with a 36‐mm cylindrical probe. The probe was positioned at a rate of 1 mm per second, descending to 30 mm below the surface of the mayonnaise in the container, and then it was returned to its original position (Ünver and Çelik 2023).

### 2.10 Sensory Evaluation

Taste, odor, consistency, appearance, mouthfeel, and overall acceptability characteristics of mayonnaise samples were assessed by 25 semi‐trained panelists without egg allergy (18–44 years old, 13 female, and 12 male) on a nine‐point scale (1: *most disliked*, 5: *neither liked nor disliked*, 9: *most liked*). Samples labeled with three‐digit numbers were served on ceramic plates with breadsticks and drinking water to clear the palate (Ünver and Çelik 2023). The sensory analysis of the present study was approved by the Science and Engineering Sciences Scientific Research Ethics Committee of Necmettin Erbakan University (E‐66991687‐100‐681947‐2025/07).

### 2.11 Statistical Analysis

Each experiment was conducted a minimum of three times, and the results are reported as the mean along with the standard deviation. One‐way ANOVA and Tukey HSD test were performed through JMP 5.0 (SAS, North Carolina, USA) software with a significance level of 0.05.

## 3 Results and Discussion

### 3.1 Color Values of Mayonnaise Samples

The color of foods plays a crucial role in the evaluation of quality, providing the initial visual experience for consumers. Personal interpretations of color vary and can play a significant role in the acceptance of a food product (Çölük et al. 2025). Color values of mayonnaise samples containing flaxseed mucilage are given in Table 1.

**TABLE 1:** Color values of mayonnaise samples.

Sample	L*	a*	b*	SI	Hue	Δ*E*
100% EY	78.34 ± 0.86^c^	−2.14 ± 0.44^a^	19.62 ± 0.88^a^	19.74 ± 0.69^a^	96.21 ± 0.94^de^	—
75% EY	80.86 ± 0.78^abc^	−2.51 ± 0.47^a^	16.13 ± 0.76^b^	16.32 ± 0.74^b^	98.84 ± 0.80^cd^	4.32 ± 0.80^d^
50% EY	82.42 ± 0.74^ab^	−2.84 ± 0.51^a^	12.32 ± 0.82^c^	12.64 ± 0.87^c^	102.98 ± 0.86^b^	8.40 ± 0.74^bc^
25% EY	83.77 ± 0.85^a^	−3.02 ± 0.48^a^	9.43 ± 0.76^cd^	9.90 ± 0.82^cd^	107.76 ± 0.72^a^	11.58 ± 0.82^a^
75% EY:25% FM	80.37 ± 0.75^bc^	−2.19 ± 0.50^a^	16.00 ± 0.83^b^	16.15 ± 0.91^b^	97.78 ± 0.77^cd^	4.15 ± 0.73^d^
50% EY:50% FM	83.83 ± 0.72^a^	−1.22 ± 0.59^a^	17.22 ± 0.71^ab^	17.26 ± 0.86^ab^	94.05 ± 0.83^e^	6.06 ± 0.87^cd^
25% EY:75% FM	81.21 ± 0.93^abc^	−1.88 ± 0.47^a^	9.91 ± 0.76^cd^	10.08 ± 0.80^cd^	100.75 ± 0.71^bc^	10.13 ± 0.80^ab^
100% FM	78.16 ± 0.89^c^	−1.34 ± 0.51^a^	7.51 ± 0.93^d^	7.63 ± 0.72^d^	100.12 ± 0.80^bc^	12.14 ± 0.76^a^

*Note*: Means with different superscript letters in the same column are significantly (*p* < 0.05) different.

Abbreviations: EY, egg yolk; FM, flaxseed mucilage; SI, saturation index; ΔE, total color difference.

Compared to the control mayonnaise made from 100% egg yolk, the use of flaxseed mucilage at a 100% level elicited similar L* and a* values (Table 1). However, b* and *SI* values of samples decreased with the substitution of 75% and 100% of egg yolk with mucilage. On the other hand, mayonnaises made from 75% and 100% flaxseed mucilage had greater *hue* and Δ*E* values. At the same egg yolk levels, mayonnaise samples made with flaxseed mucilage showed similar L* and a* values to the negative controls. According to Herald et al. (2009), changes in the color values of mayonnaise samples were due to the yellow pigments (carotene, lutein, xanthophylls, cryptoxanthin, etc.) found in egg yolk, not being present in the egg replacers. They also obtained similar results when replacing egg yolk with modified corn starch.

### 3.2 pH and Moisture Content

The pH changes in mayonnaise samples are demonstrated in Table 2. The pH value of mayonnaise made from 25% flaxseed mucilage (4.01) was similar to the control (4.07). However, higher levels (> 25%) of flaxseed mucilage decreased the pH of mayonnaise, and the lowest pH (3.58) was obtained with the substitution of 100% of egg yolk by mucilage. Flaxseed mucilage, which is a complex polysaccharide, consists of neutral (xylose, arabinose, and galactose) and acidic (rhamnose, glucose, fucose, and galacturonic acid) fractions (Yu et al. 2022). Acidic fractions in flaxseed mucilage could be responsible for the decrease in the pH of mayonnaise. Ali and el Said (2020) also found a decrease in pH values of mayonnaise by replacing egg yolk with gum Arabic. *Salmonella* contamination in mayonnaise is still a troubling issue for public health. The low pH values of mucilage‐containing samples can provide an advantage to reduce the risk of microbiological spoilage.

**TABLE 2:** pH and moisture values and stability properties of mayonnaise samples.

Sample	pH	Moisture (%)	Emulsion stability (%)	Free fatty acids (% oleic acid)	Peroxide value (meqO_2_/kg)
100% EY	4.07 ± 0.04^a^	16.28 ± 0.10^d^	100.0 ± 0.0^a^	0.97 ± 0.01^a^	3.32 ± 0.12^a^
75% EY	4.02 ± 0.03^ab^	16.03 ± 0.07^d^	95.6 ± 1.0^b^	0.98 ± 0.03^a^	3.33 ± 0.10^a^
50% EY	3.90 ± 0.04^bc^	15.68 ± 0.08^e^	87.3 ± 1.3^c^	1.00 ± 0.01^a^	3.36 ± 0.14^a^
25% EY	3.79 ± 0.01^cd^	15.24 ± 0.06^f^	70.6 ± 1.0^d^	1.02 ± 0.03^a^	3.37 ± 0.16^a^
75% EY:25% FM	4.01 ± 0.03^ab^	18.73 ± 0.08^c^	100.0 ± 0.0^a^	0.80 ± 0.03^b^	2.78 ± 0.15^b^
50% EY:50% FM	3.87 ± 0.03^c^	19.65 ± 0.07^b^	100.0 ± 0.0^a^	0.77 ± 0.01^b^	2.20 ± 0.11^c^
25% EY:75% FM	3.74 ± 0.01^d^	19.69 ± 0.09^b^	97.0 ± 1.4^ab^	0.75 ± 0.00^b^	2.01 ± 0.16^c^
100% FM	3.58 ± 0.04^e^	21.87 ± 0.08^a^	99.4 ± 1.1^a^	0.76 ± 0.02^b^	1.73 ± 0.13^c^

*Note*: Means with different superscript letters in the same column are significantly (*p* < 0.05) different.

Abbreviations: EY, egg yolk; FM, flaxseed mucilage.

As can be seen in Table 2, the moisture content of mayonnaise samples ranged between 15.24% and 21.87%. In negative control groups, reducing the egg yolk level from 100% to 25% reduced the moisture content from 16.28% to 15.24% of mayonnaise, while replacing egg yolk with flaxseed mucilage considerably increased the moisture content. The increase in the moisture content may be due to the use of flaxseed mucilage in aqueous form in mayonnaise formulations. These findings align with the results of a previous study by Cornelia et al. (2015), who investigated the impact of replacing egg yolk with durian seed gum on the mayonnaise quality.

### 3.3 Emulsion Stability

Emulsion stability plays a vital role in the quality of mayonnaise and is influenced by the size and consistency of oil droplets, type and concentration of emulsifier, pH, viscosity, process temperature, and method (Amrinola and Zeafitri 2025). The emulsion stability of mayonnaise samples is shown in Table 2. There were no significant (*p* < 0.05) differences between the control and mucilage‐containing mayonnaise samples. The results may be associated with the high emulsification ability of flaxseed mucilage (Yu et al. 2022), which helps protect the droplets against destabilization, despite the reduced protein content of mayonnaise by replacing egg yolk (Cornelia et al. 2015). Besides that, negative controls had lower emulsion stability than the control 100% egg mayonnaise and mucilage‐added samples (Table 2). The findings revealed that flaxseed mucilage has a strong emulsifying capacity and can be used as an egg replacer. Herald et al. (2009) observed similar behavior in mayonnaise made with fenugreek seed gum as an egg replacer. Wang et al. (2022) also reported that xanthan gum exhibited a positive impact on the emulsion stability of mayonnaise.

### 3.4 Oxidative Stability

Lipid oxidation causes adverse alterations in emulsions, resulting in deteriorated sensory and nutritional quality in food products. Strong stability has a positive contribution to the extension of the shelf life of mayonnaise. To evaluate the oxidative stability of mayonnaise samples, FFA content and peroxide value results are presented in Table 2.

FFA contents of mayonnaise samples changed between 0.75% and 1.02% (Table 2). The highest FFA content was obtained in the control and negative controls. The replacement of egg yolk with flaxseed mucilage notably decreased the FFA content of mayonnaise samples. Although egg yolk contains high amounts of lipids (32.13%) (Sun et al. 2013), flaxseed mucilage has poor lipid content (5.07%) (Yaver 2026). Therefore, the replacement of egg yolk with flaxseed mucilage can reduce the oil content of mayonnaise (Fernandes and Mellado 2018), resulting in a decrease in the risk of lipid oxidation as well as FFA content (Rojas‐Martin et al. 2023).

As shown in Table 2, the greatest peroxide value was obtained in the control and negative controls. The replacement of egg yolk with flaxseed mucilage considerably decreased the peroxide value of mayonnaise samples compared to the control and negative controls. According to Jacobsen et al. (2001), the low pH content of mayonnaise could cause the release of iron from egg yolk as a result of the breaking of iron bridges between low‐density lipoproteins, phosvitin, and lipovitellins. The increasing iron level can activate the oxidative enzymes and accelerate oxidation reactions in mayonnaise. Moreover, Büyük et al. (2024) suggested that the addition of hydrocolloids can improve the oxidative stability of mayonnaise by decreasing lipid content. The decreasing lipid content of mayonnaise by the replacement of egg yolk with flaxseed mucilage (Fernandes and Mellado 2018) may decrease the generation of peroxides. Therefore, the replacement of egg yolk with flaxseed mucilage as an iron‐ and lipid‐poor emulsifier might improve the oxidative stability of mayonnaise (Rojas‐Martin et al. 2023). On the other hand, flaxseed mucilage contains a large amount of bioactive compounds such as ferulic acid, *p*‐coumaric acid, and gallic acid, which have strong antioxidant activity (Yu et al. 2022; Kucka et al. 2024). The high antioxidant activity of flaxseed mucilage could be responsible for the lower peroxide values of mucilage‐containing mayonnaises (Ali and el Said 2020). Hijazi et al. (2022) investigated the oxidative stability of vegan mayonnaise formulated with flaxseed mucilage as a fat replacer. They stated that flaxseed mucilage can show strong oxidative stability with high consistency in vegan mayonnaise. The data obtained in the present study highlighted that flaxseed mucilage could provide higher oxidative stability in mayonnaise as an egg yolk replacer.

### 3.5 Microbial Stability

The quality of mayonnaises was assessed microbiologically, and the data are presented in Table 3. The results revealed that TAMB and yeast/mold counts for all mayonnaise samples were found to be less than 10 CFU/g. Furthermore, coliforms were not detected in mayonnaise samples. These results may be associated with the low pH values of mayonnaise samples (Table 2). In a recent study by Kucka et al. (2024), the antimicrobial activity of flaxseed mucilage against Gram‐positive bacteria (*Bacillus subtilis, Enterococcus faecalis*, and *Staphylococcus aureus*), Gram‐negative bacteria (*S. enterica, Yersinia enterocolitica*, and *Pseudomonas aeruginosa*), and yeasts (*Candida albicans, C. glabrata, C. krusei*, and *C. tropicalis*) was investigated. They found that flaxseed mucilage has a strong antimicrobial activity against the tested microorganisms. Ali and el Said (2020) evaluated the use of gum Arabic as an egg replacer in vegan mayonnaise. The authors stated that gum Arabic elicited a similar microbiological quality to the control.

**TABLE 3:** Microbial counts of mayonnaise samples.

Sample	TAMB (CFU/g)	Yeast and molds (CFU/g)	Coliforms (CFU/g)
100% EY	<10	<10	nd
75% EY	<10	<10	nd
50% EY	<10	<10	nd
25% EY	<10	<10	nd
75% EY:25% FM	<10	<10	nd
50% EY:50% FM	<10	<10	nd
25% EY:75% FM	<10	<10	nd
100% FM	<10	<10	nd

Abbreviations: EY, egg yolk; FM, flaxseed mucilage; TAMB, total aerobic mesophilic bacteria; CFU, colony forming units; nd, not detected.

### 3.6 Textural Properties

The results of evaluating the impact of replacing egg yolk with flaxseed mucilage on the textural properties (hardness, adhesiveness, cohesiveness, and gumminess) of mayonnaise samples are demonstrated in Table 4.

**TABLE 4:** Textural properties of mayonnaise samples.

Sample	Hardness (N)	Adhesiveness (N.sn)	Cohesiveness (N)	Gumminess (N)
100% EY	1.58 ± 0.05^b^	1.90 ± 0.05^ab^	0.01 ± 0.00^a^	1.45 ± 0.03^a^
75% EY	1.42 ± 0.04^b^	1.75 ± 0.03^b^	0.01 ± 0.00^a^	1.25 ± 0.04^b^
50% EY	1.03 ± 0.03^cd^	1.14 ± 0.04^c^	0.01 ± 0.00^a^	0.91 ± 0.06^c^
25% EY	0.45 ± 0.06^f^	0.41 ± 0.03^e^	0.01 ± 0.00^a^	0.58 ± 0.04^d^
75% EY:25% FM	1.79 ± 0.07^a^	1.91 ± 0.04^a^	0.01 ± 0.00^a^	1.54 ± 0.05^a^
50% EY:50% FM	1.18 ± 0.03^c^	1.29 ± 0.02^c^	0.01 ± 0.00^a^	1.01 ± 0.05^c^
25% EY:75% FM	0.81 ± 0.04^e^	0.69 ± 0.05^d^	0.01 ± 0.00^a^	0.69 ± 0.04^d^
100% FM	0.84 ± 0.05^de^	0.54 ± 0.04^de^	0.01 ± 0.00^a^	0.67 ± 0.03^d^

*Note*: Means with different superscript letters in the same column are significantly (*p* < 0.05) different. EY, egg yolk; FM, flaxseed mucilage.

Hardness shows the strength necessary to squish food between the molar teeth (Raikos et al. 2020). Compared to the control and negative controls, the replacement of egg yolk by 25% of flaxseed mucilage increased the hardness of mayonnaise (Table 4). The use of flaxseed mucilage may improve the protein–polysaccharide interaction. The oil droplets in mayonnaise could be surrounded by protein–polysaccharide interactions, resulting in a strong network and a harder texture (Aalami et al. 2023). However, increasing levels of mucilage elicited lower hardness values in mayonnaise compared with the control (Table 4). The microstructure of mayonnaise is affected by several factors, including emulsifier type and concentration, viscosity, oil content, and droplet size (Laca et al. 2010). Fernandes and Mellado (2018) observed that an increasing level of chia mucilage as an egg replacer decreased the viscosity index of mayonnaise. Decreasing the protein and phospholipid content of mayonnaise by replacing egg yolk with polysaccharide‐rich flaxseed mucilage (Cornelia et al. 2015) may reduce the viscosity and enlarge droplet size, further resulting in lower hardness values in mayonnaise (Table 4). Herald et al. (2009) also found a decrease in firmness values of mayonnaise samples when replacing egg yolk with modified corn starch. On the other hand, mayonnaise made with 75% and 100% flaxseed mucilage elicited a harder texture than mayonnaise made with 25% egg yolk (Table 4). The data showed the effectiveness of flaxseed mucilage as an egg replacer on the texture of mayonnaise.

Adhesiveness indicates the amount of energy needed to remove mayonnaise from a knife or spoon (Raikos et al. 2020). As seen in Table 4, mayonnaise prepared from 25% flaxseed mucilage had similar adhesiveness to that made by 100% egg yolk, and higher than the negative controls. Conversely, the adhesiveness of samples decreased as the mucilage level increased from 25% to 100%. The lowering of viscosity values due to the addition of flaxseed mucilage may be responsible for the lower adhesiveness values in mayonnaise (Nikzade et al. 2012). According to Jing et al. (2023), a decrease in adhesiveness of mayonnaise is advantageous, allowing it to stick less to the knife and facilitating easier spreading, because it is commonly used as a spreadable product.

Cohesiveness measures the intensity of the internal links in mayonnaise and the level of deformation it can withstand before failure (Raikos et al. 2020). The results demonstrated that the replacement of egg yolk with flaxseed mucilage at all levels did not notably affect the cohesiveness of mayonnaise (Table 4).

In terms of the gumminess parameter, mayonnaise made with 25% mucilage demonstrated similar results to the control (Table 4). However, mucilage substitution for egg yolk at high levels elicited lower gumminess values in mayonnaise than those of the control. The softer texture of these samples may be responsible for the lower gumminess values.

### 3.7 Sensory Quality

**FIGURE 1:**
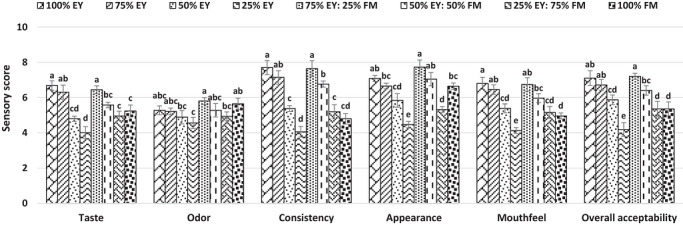
Sensory evaluation of mayonnaise samples.

Sensory evaluation of mayonnaise samples is shown in Figure 1. The taste score of mayonnaise made with 25% flaxseed mucilage was close to the control. However, increasing levels of flaxseed mucilage decreased the taste scores of mayonnaise. The decrease may be related to their lower pH values than those of the control (Table 2), which may lead to the release of sour taste (X. Liu et al. 2018). Besides that, taste scores of mayonnaise prepared with 75% and 100% flaxseed mucilage were higher than the negative control containing 25% egg yolk (Figure 1). On the other hand, there is no considerable difference in odor scores between the control mayonnaise and flaxseed mucilage‐containing mayonnaises, probably due to the strong smell of vinegar. The sample made with 25% flaxseed mucilage had a similar consistency score to the control and negative control containing 75% egg yolk. The replacement of egg yolk with 75% and 100% of flaxseed mucilage decreased the consistency scores of mayonnaise samples. These results are consistent with the results of textural analysis (Table 4). Mayonnaise samples produced with 25%, 50%, and 100% of flaxseed mucilage had comparable appearance scores to the control (Figure 1). In terms of mouthfeel parameter, mayonnaise samples formulated with 25% and 50% mucilage received similar scores to the control, and higher scores than negative controls. However, high levels of flaxseed mucilage decreased mouthfeel scores of mayonnaise compared to the control. Changes in the textural properties of mayonnaise samples because of the addition of high levels of mucilage (Table 4) might be responsible for the lower mouthfeel scores (Herald et al. 2009). The highest overall acceptability scores were obtained in the control, negative control made with 75% egg yolk and 25% flaxseed mucilage‐containing sample (Figure 1). In addition, 75% and 100% mucilage‐added samples had greater scores than the negative control made with 25% egg yolk. Odep et al. (2024) found a strong correlation between the taste and overall acceptability scores of mayonnaise samples. Therefore, the low taste scores of mayonnaise made with 75% and 100% of flaxseed mucilage may have influenced the overall acceptability scores (Figure 1).

## 4 Conclusion

In this study, egg yolk was replaced with flaxseed mucilage in mayonnaise formulation, aiming at the development of healthy products. The findings showed that high levels (75% and 100%) of flaxseed mucilage considerably changed the color values of mayonnaise. Compared to the control, the increasing levels of mucilage decreased the pH value and increased the moisture content of the mayonnaise samples. Emulsion stability of mucilage‐added samples was similar to that of the control. Besides that, replacement of egg yolk with flaxseed mucilage elicited superior emulsion stability than negative controls. The use of flaxseed mucilage as an egg replacer positively influenced the oxidative stability of mayonnaise in comparison with the control and negative control samples. In terms of microbial quality, flaxseed mucilage revealed good stability. Compared with the control, the use of flaxseed mucilage (> 25%) showed a softer texture and lower adhesiveness. The replacement of egg yolk with flaxseed mucilage elicited acceptable scores for overall acceptability. This research highlighted the potential of flaxseed mucilage as a sustainable egg replacer in developing healthy mayonnaise formulations with good oxidative and microbial stability.

## Author Contributions

**Elif Yaver**: investigation, resources, methodology, formal analysis, conceptualization, writing – original draft, writing – review and editing. **Eda Güneş**: methodology, formal analysis, conceptualization, writing – review and editing. **Nursena Adıyaman**: investigation, resources, formal analysis.

## Conflicts of Interest

The authors declare no conflicts of interest.

## Data Availability

Data are only available upon request for corresponding author.
